# Antimicrobial and cleaning effects of ultrasonic-mediated plasma-loaded microbubbles on *Enterococcus faecalis* biofilm: an in vitro study

**DOI:** 10.1186/s12903-023-02813-6

**Published:** 2023-03-08

**Authors:** Mengqian Zhu, Jie Dang, Feihong Dong, Ruoqing Zhong, Jue Zhang, Jie Pan, Yinglong Li

**Affiliations:** 1 Department of General Dentistry, Peking University School and Hospital of Stomatology, National Center of Stomatology, National Clinical Research Center for Oral Diseases, National Engineering Research Center of Oral Biomaterials and Digital Medical Devices, Beijing, 100081 China; 2 Academy for Advanced Interdisciplinary Studies, Peking University, Beijing, 100871 China; 3 College of Engineering, Peking University, Beijing, 100871 China; 4 Department of Stomatology, Beijing Chao-Yang Hospital, Capital Medical University, Beijing, 100020 China

**Keywords:** *Enterococcus faecalis* biofilm, Plasma-loaded microbubbles (PMBs), Ultrasound treatment, Endodontic treatment, Mechanical safety

## Abstract

**Background:**

*Enterococcus faecalis* (*E. faecalis*) is the most frequently isolated bacteria from teeth with root canal treatment failure. This study aims to evaluate the disinfection effect of ultrasonic-mediated cold plasma-loaded microbubbles (PMBs) on 7d *E. faecalis* biofilm, the mechanical safety and the mechanisms.

**Methods:**

The PMBs were fabricated by a modified emulsification process and the key reactive species, nitric oxide (NO) and hydrogen peroxide (H_2_O_2_) were evaluated. The 7d *E. faecalis* biofilm on human tooth disk was constructed and divided into the following groups: PBS, 2.5%NaOCl, 2%CHX, and different concentrations of PMBs (10^8^ mL^−1^, 10^7^ mL^−1^). The disinfection effects and elimination effects were verified with confocal laser scanning microscopy (CLSM) and scanning electron microscopy (SEM). Microhardness and roughness change of dentin after PMBs treatment were verified respectively.

**Results:**

The concentration of NO and H_2_O_2_ in PMBs increased by 39.99% and 50.97% after ultrasound treatment (*p* < 0.05) respectively. The CLSM and SEM results indicate that PMBs with ultrasound treatment could remove the bacteria and biofilm components effectively, especially those living in dentin tubules. The 2.5% NaOCl presented an excellent effect against biofilm on dishes, but the elimination effect on dentin tubules is limited. The 2% CHX group exhibits significant disinfection effect. The biosafety tests indicated that there is no significant changes on microhardness and roughness after PMBs with ultrasound treatment (*p* > 0.05).

**Conclusion:**

PMBs combined with ultrasound treatment exhibited significant disinfection effect and biofilm removal effect, the mechanical safety is acceptable.

## Background

The persistent infection in the root canal is the primary cause of root canal treatment failure. Many studies have demonstrated that *Enterococcus faecalis* (*E. faecalis*) is the most dominant species associated with persistent infection owing to their great resistance capacity and biofilm formation ability [1]. Root canal disinfection control is supposed to be accomplished with mechanical instruments and chemical irrigations. Traditional chemical irrigations, such as sodium hypochlorite (NaOCl) and chlorhexidine (CHX), had been widely used in clinic. However, the traditional methods still have some limitations, due to the narrow space and complex anatomical structure of the root canal together with the innate resistance capacity of microorganisms [2, 3]. More important, there are 20,000–45,000 dentin tubules per square millimeter, the dentin tubules were also infected in most cases and posed great potential to lead root canal treatment failure. To attain better disinfection and debridement effects, some novel methods have been reported, such as laser [4, 5], photodynamic therapy (PDT) [6, 7] and ozone [8, 9]. Several root canal irrigations like super-oxidized water [10], QMix [11, 12] and MTAD [13] had also been reported. However, there still exist some limitations, such as incomplete absorption of laser, limited distribution of photosensitizers in canal system, extremely short effective time of active oxygen components, orange-brown precipitate formed and insufficient antibacterial performance [14]. Meanwhile, the dead bacteria and biofilm matrix remained in root canals, which might act as potential source of infection and lead to root canal treatment failure.

Cold atmosphere pressure plasma (cold plasma), as a partially ionized gas containing charged ions and free radicals, has various medical and biomedical applications involving blood coagulation [15], wound healing [16] and disinfection [17, 18]. Our previous study had demonstrated that cold plasma shows a great antibacterial effect against *E. faecalis* biofilm owing to its abundant reactive substances [19]. However, the dosage of cold plasma was difficult to quantify. Meanwhile, microbubbles have been widely applied in diagnostic medicine as ultrasound contrast agents to enhance ultrasound imaging. Recently, it has drawn great attention in the field of drug delivery for its cavitation when exposed to ultrasound [20]. The characteristic endows microbubbles with capacity to delivery and release drug at the target site through narrow spaces. Some studies also have investigated its potential applications in endodontic disinfection, the microbubbles combined with ultrasound can effectively reduce bacteria and strengthen the efficiency of disinfecting agents [21, 22]. Compared with planktonic bacteria, biofilm could even exhibit 10–1000 times of antibiotic resistance.

Above all, to remove the biofilm on dentin surface, a novel plasma-loaded microbubbles (PMBs) were fabricated, which were expected to package the antibacterial reactive species of cold plasma, and to be released immediately after the cavitation effect of ultrasonic. This precise treatment is a superior treatment to eliminate endodontic infection. The aim of this study is to investigate the disinfection and removal effects of PMBs combined with ultrasound on the 7d of *E. faecalis* biofilm and the biosafety of the treatment in vitro. The null hypothesis: (1) PMBs with ultrasound are not expected to significantly reduce the number of viable bacterial cells compared to the other treatment groups tested. (2) No significant changes in microhardness and roughness compared to the other treatment groups tested are expected after the application of PMBs combined with ultrasound treatment.

## Methods

### Preparation and characterization of plasma-loaded microbubbles (PMBs)

**Fig. 1:**
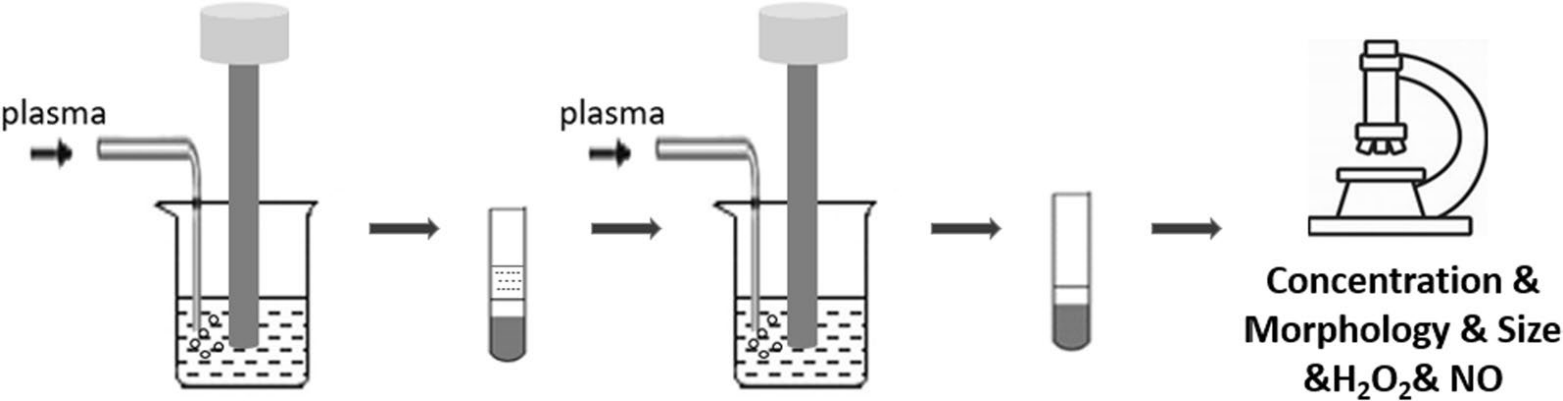
Schematic illustration of the preparation and characterization of plasma loaded microbubbles (PMBs)

PMBs were fabricated by a modified emulsification process as described in previous study [23]. The mixture of Span 60, NaCl, Tween 80, PBS and polyethylene glycol (PEG-4000) was stirred at room temperature and autoclaved at 121℃ for 12 min, subsequently cooled down to 40 ℃. As Fig. 1 shows, 18 mL of the suspension was sonicated for 2 min with constant purging of plasma gas for 6 s. After standing still for 3 h and separating into 3 layers, 4 mL of the middle layer were diluted in 8 mL PBS. And the total mixture was sonicated again to obtain the PMBs.

Then, the PMBs were evaluated by a light microscopy to determine the concentration with blood cell counting plate. The morphology characterization of PMBs was determined by a bright field microscope. The size distribution was measured at different time points for stability assessment using a multi-angle particle size analyzer (Brookhaven, USA). The key reactive species, nitric oxide (NO) and hydrogen peroxide (H_2_O_2_) existed in PMBs suspension and release levels after ultrasound sonication were measured with a corresponding assay kit according to the manufacturer’s instructions (Beyotime, China), respectively. The values for the control group were obtained with PBS solution.

### Preparation of dentin specimens

**Fig. 2:**
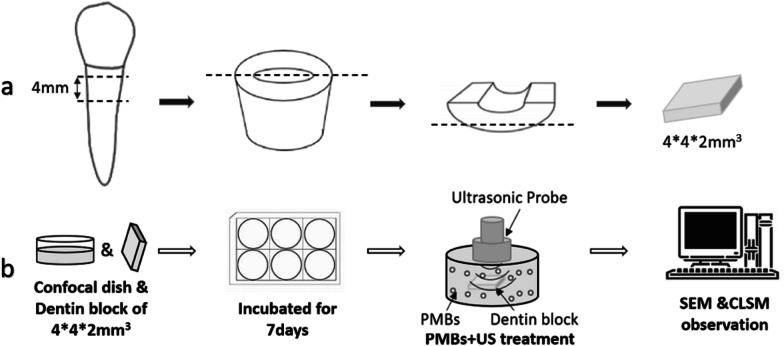
The schematic illustration of the preparation of dentin blocks (**a**) and the in vitro study (**b**)

This study was approved by the bioethics committee of Beijing Chaoyang Hospital of Capital Medical University (Registration number: 2020-ke-28). The single-rooted, caries free human teeth were collected and stored in 0.01% thymol solution at 4 ℃. As Fig. 2a shows, the preparation process was carried out refer to Shen Ya [24]. Fifteen teeth were selected and sectioned at 1 mm below the cementoenamel junction (CEJ) to a unified length of 4 mm cylindrical dentin block. The root segments were enlarged to F3 and each dentin block was grinded longitudinally into 2 pieces. Each tooth disk was subsequently shaped into approximately 4*4*2 mm. All samples were cleaned by using ultrasound in 2.5% NaOCl and 17% EDTA for 2 min respectively. Finally, normal saline was used to clean and sterilized by autoclaving (121 °C) for 15 min before use.

### Cultivation of *E. faecalis* biofilm

The bacterial strain used in this study was *E. faecalis* (ATCC 29212). The inoculation and culture were performed as previous study [25]. As Fig. 2b shows, 100μL of *E. faecalis* suspension and 1 mL sterile brain heart infusion (BHI) broth was added into a confocal dish (or with prepared dentin block) and incubated in an aerobic environment for 7 days, and the BHI broth was replaced every 48 h. After incubation for 7 d, all samples were washed three times with sterile PBS to remove the unattached or loosely attached bacteria. As Table 1 shows, 7d *E. faecalis* biofilm samples were divided into 5 groups randomly. The PMBs were diluted to 10^7^ mL^−1^ and 10^8^ mL^−1^ before use. The ultrasound setting parameters were 3W/cm^2^ acoustic intensity, 1 MHz frequency, and 50% duty cycle (Sonic-Stimu Pro, NU-TEK Health Ltd, Hong Kong, China). All samples were analyzed immediately after treatment.

**Table 1:** Groups of different treatment

Group	Treatment	Group	Treatment
A	PBS	D	PMBs (10^7^ mL^−1^) + US
B	2.5% NaOCl	E	PMBs (10^8^ mL^−1^) + US
C	2% CHX		

### Confocal laser scanning microscopy analysis

To investigate the antimicrobial effect of PMBs, the *E. faecalis* biofilm was incubated on the coverslips. Next, the antimicrobial effect was determined by confocal laser scanning microscopy (CLSM, A1R-si, Nikon, Japan) with the excitation/emission wavelengths 488 nm/543 nm. According to the manufacturer’s instructions, the samples were stained with the LIVE/DEAD Baclight™ Bacterial Viability Kit (Invitrogen, Carlsbad, CA) for 15 min in dark environment and then rinsed slightly with PBS for three times. Simultaneous dual-channel imaging was used to display green fluorescence (viable cells) and red fluorescence (dead cells) after treatment. The mounted samples were observed by using a × 20 lens and imaged with the Zen Blue Edition software (Zeiss, Germany).

### Scanning electron microscopy

Scanning electron microscopy (SEM, S-4800, HITACHI, Japan) was used to observe the surface morphology and structural changes. The treated specimens were immersed with 2.5% glutaraldehyde at 4 ℃ for 48 h and subsequently dehydrated in a series of ethanol solutions. After dehydration, the specimens were dried at 50 ℃ overnight for examination.

### Mechanical safety test

The mechanical safety was tested including roughness and microhardness changes of dentin before and after treated with PMBs + US. Ten prepared dentin blocks were embedded in epoxy resin at room temperature and the pupal wall was gradient grinded with #800, #1000, #1500, #2000, #2500, #3000, #4000 with grinding paper under running wate. All samples were randomly divided into two groups for microhardness and roughness test. Roughness (Ra, μm) was tested by 3D Profile Measurement Laser Microscope (VK-X200, Keyence Corp., Japan). Meanwhile Vickers Hardness Tester (VHN) (Shimadzu Corp., Japan) was used for microhardness test with a 300 g load and a dwelling time of 10 s.

### Statistical analysis

The statistical significance was calculated by using SPSS version 22.0. One-way analysis of variance (ANOVA) was conducted to compare the difference of diameter between the four groups. Moreover, the paired-sample *t*-test was applied for reactive substances content and mechanical safety tests. And the statistical significance was considered at *p* < 0.05.

## Results

### Physicochemical characterization of PMBs

**Fig. 3:**
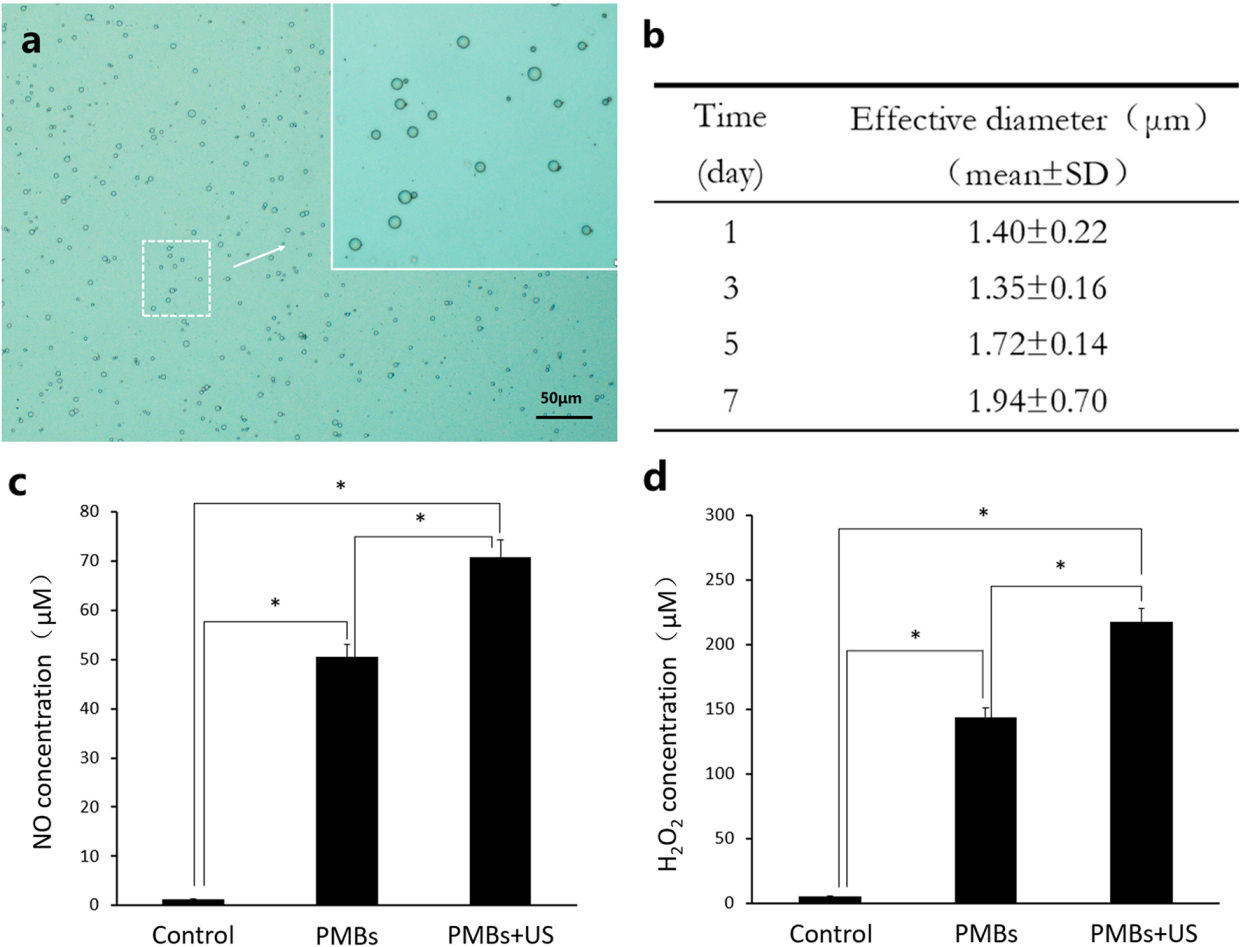
**a** Light microscopic images of PMBs. **b** The effective diameter of PMBs at 1d, 3d, 5d and 7d. **c**, **d** Comparison of NO, H_2_O_2_ concentrations in PMBs with and without ultrasound treatment (*p* < 0.05)

Microbubbles are usually micrometer sized (1–8 μm) structures with a gas core stabilized by a compressible shell. As shown in Fig. 3a, the core–shell structure of PMBs can be clearly identified, which consisted of a bright gaseous plasma core encapsulated in a dark surfactant shell. The size of microbubbles is one of the essential characteristics which enable them to penetrate into small spaces for their widespread clinical application. As shown in Fig. 3b, we measured the diameter at different time points (1d, 3d, 5d, 7d) for stability assessment. The effective diameter of the PMB was 1.40 ± 0.22 μm. And with the increase of storage time, the diameter of PMBs appeared to a trend of increase but stabilized at about 1–2 μm (*p* > 0.05).

As shown in Fig. 3c, the nitric oxide (NO) concentration of the PMB group was 50.56 μmol L^−1^ and significantly increased compared with that of control group (1.27 μmol L^−1^), which was due to the plasma dissolved in solution. Additionally, compared with sole PMBs suspension, the nitric oxide concentration further increased remarkably after ultrasonic stimulation with a concentration of 70.78 μmol L^−1^. Hydrogen peroxide (H_2_O_2_) is another highly reactive species in plasma and has been found to be a powerful disinfectant. As Fig. 3d shows, we found out a similar result in the aspect of H_2_O_2_ concentration. Compared with the negligible concentration of the control group (5.14 μmol L^−1^), a remarkable increase of H_2_O_2_ concentration was observed in the PMB group (143.88 μmol L^−1^). In addition, the sonication of PMBs with ultrasound brought about a significant increase (217.22 μmol L^−1^) compared with the concentration of PMB group.

### CLSM observation

**Fig. 4:**
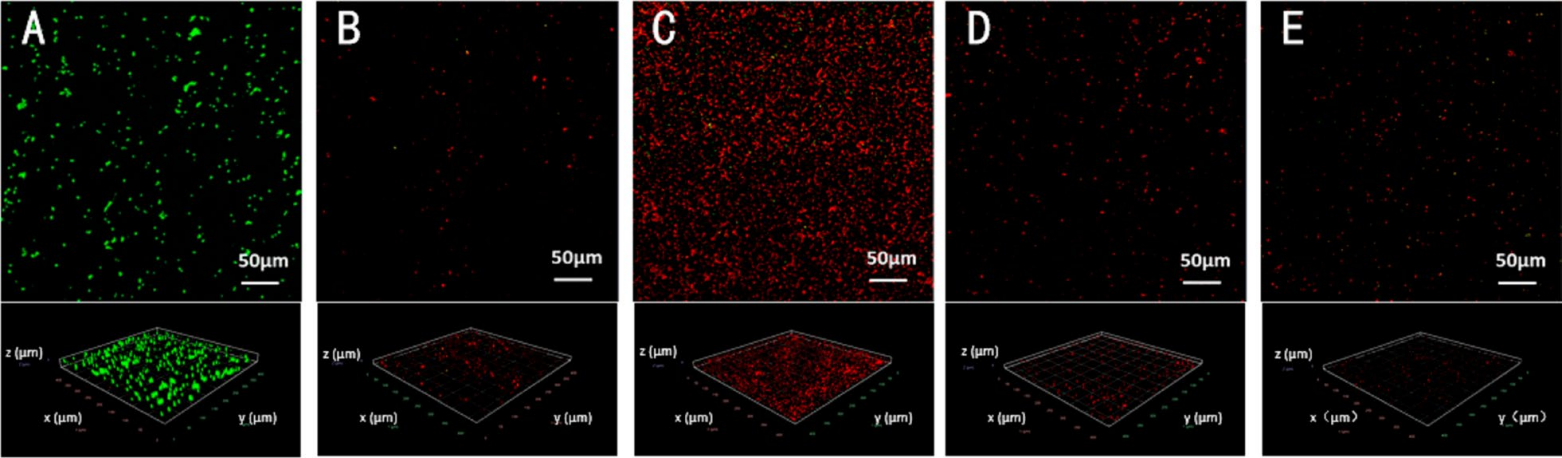
CLSM 2D and 3D images of *E. faecalis* biofilm formed on flat surface after different treatment for 5 min. **A** PBS. **B** 2.5% NaOCl. **C** 2% CHX. **D** PMBs (10^7^ mL^−1^) + US. **E** PMBs (10^8^ mL^−1^) + US

**Fig. 5:**
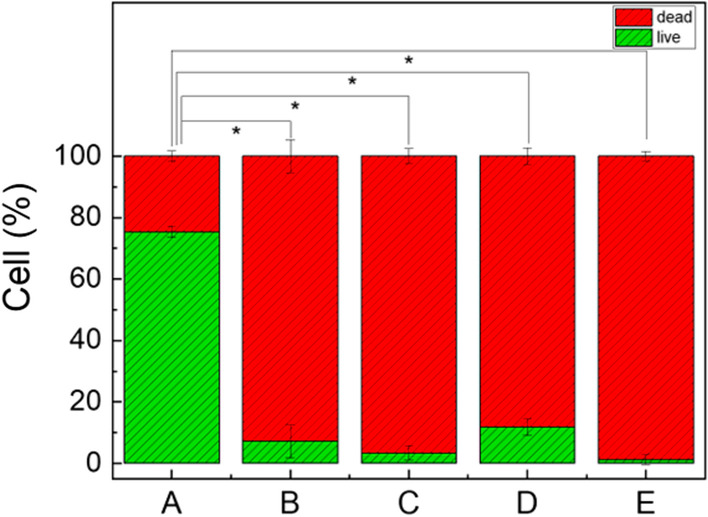
The ratio of live/dead bacteria of residual biofilm after different treatment for 5 min. **A** PBS. **B** 2.5% NaOCl. **C** 2% CHX. **D** PMBs (10^7^ mL^−1^) + US. **E** PMBs (10^8^ mL^−1^) + US

As Fig. 4 shows, the strong green fluorescence in Fig. 4A indicated that, after 7d of incubation, abundant bacteria went through growth, proliferation, and attachment to form biofilm on the bottom of the dish. The red fluorescence in Fig. 4C suggested that chlorhexidine could effectively kill *E. faecalis*, which was consistent with previous studies [26]. Additionally, the strong fluorescence signal also indicated its negligible ability to remove or dissolve biofilms. Nevertheless, in the 2.5% NaOCl group (Fig. 4B) and the two PMBs groups, the weak red fluorescence demonstrated that the majority of bacteria were eliminated. In addition, the remaining bacteria in the high concentration group (Fig. 4E, 10^8^ mL^−1^) were much cleaner than group D (Fig. 4D, 10^7^ mL^−1^). The ratio of live/dead bacteria was presented in Fig. 5, and the low ratios of group B-E were statistically different from that of the PBS group (*p* < 0.05).

### SEM observation

**Fig. 6:**
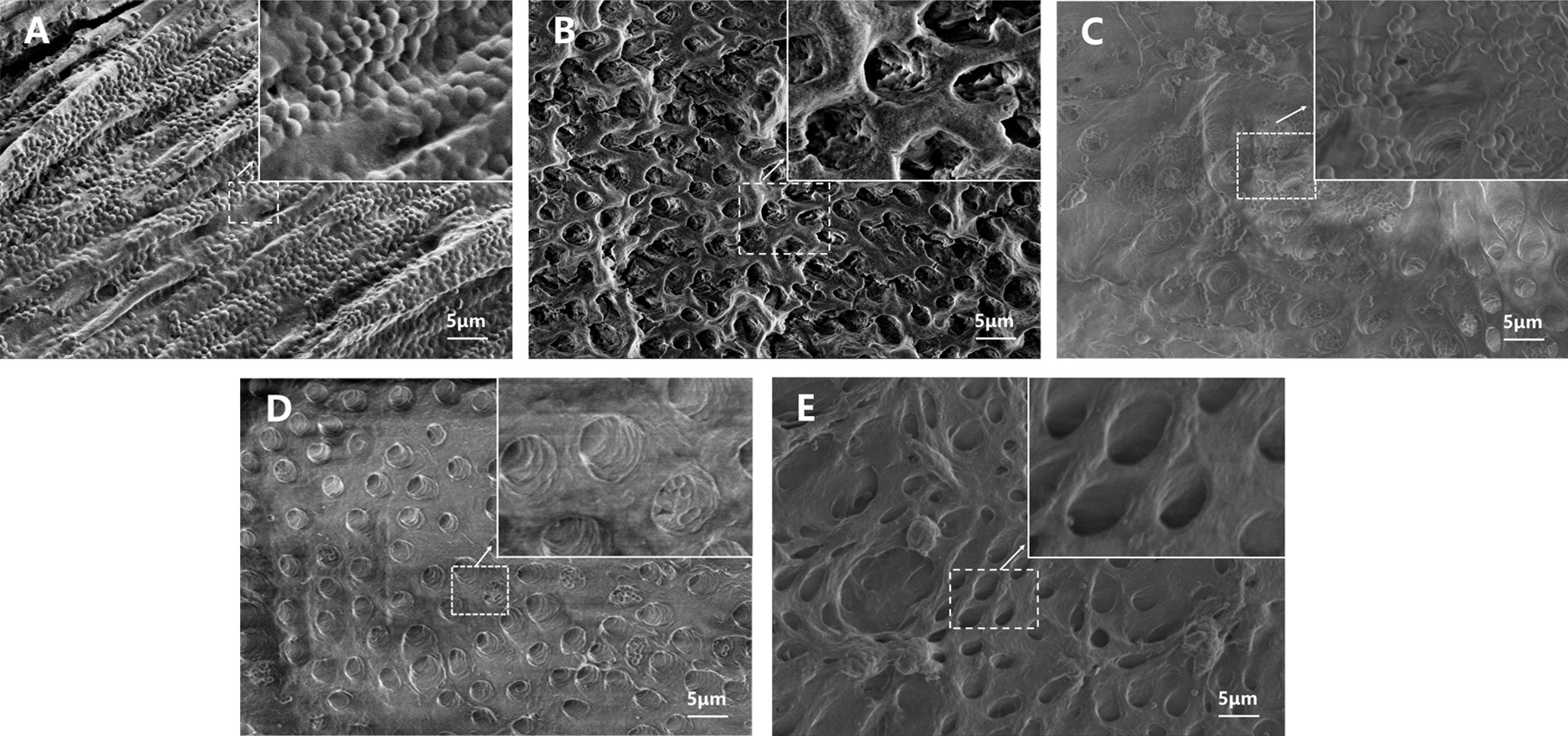
SEM images of *E. faecalis* biofilm formed on dentin surface with different treatment. **A** PBS. **B** 2.5% NaOCl. **C** 2% CHX. **D** PMBs (10^7^ mL^−1^) + US. **E** PMBs (10^8^ mL^−1^) + US

As Fig. 6A shows, dense and compact biofilm structure was observed, which indicated that abundant and integrated extracellular matrix covered on the dentin disk and the bacteria were embedded in the matrix. The 2.5% NaOCl and 2% CHX were selected as positive controls because these two are the most commonly used chemical irrigations in endodontic therapy. As Fig. 6B show, after treated with 2.5% NaOCl for 5 min, the biofilm and bacteria on the dentin were completely removed, but there existed some irregular “extracellular matrix” inside the dentin tubules. As Fig. 6C shows, the 2% CHX treatment indicated that there remained a certain amount of bacterial residue on the dentine block, especially within the dentin tubules.

Interestingly, in other two groups, which were treated with different concentrations of PMBs with ultrasound exposure, the biofilm and bacteria on the dentine block was almost removed as Fig. 6D, E show. In the low concentration group (10^7^ mL^−1^), there remained some dispersive bacteria inside the dentinal tubule. When the concentration increased (10^8^ mL^−1^), we could find out that a few “bacteria corpse” were scattered around and their spherical shape was destroyed and became crumpled and irregularly shaped. In summary, the PMBs with ultrasound stimulation could effectively remove the biofilm attached to dentin surface and dentin tubules, the structural integrity of bacteria has also been destroyed.

### Mechanical safety evaluation

**Fig. 7:**
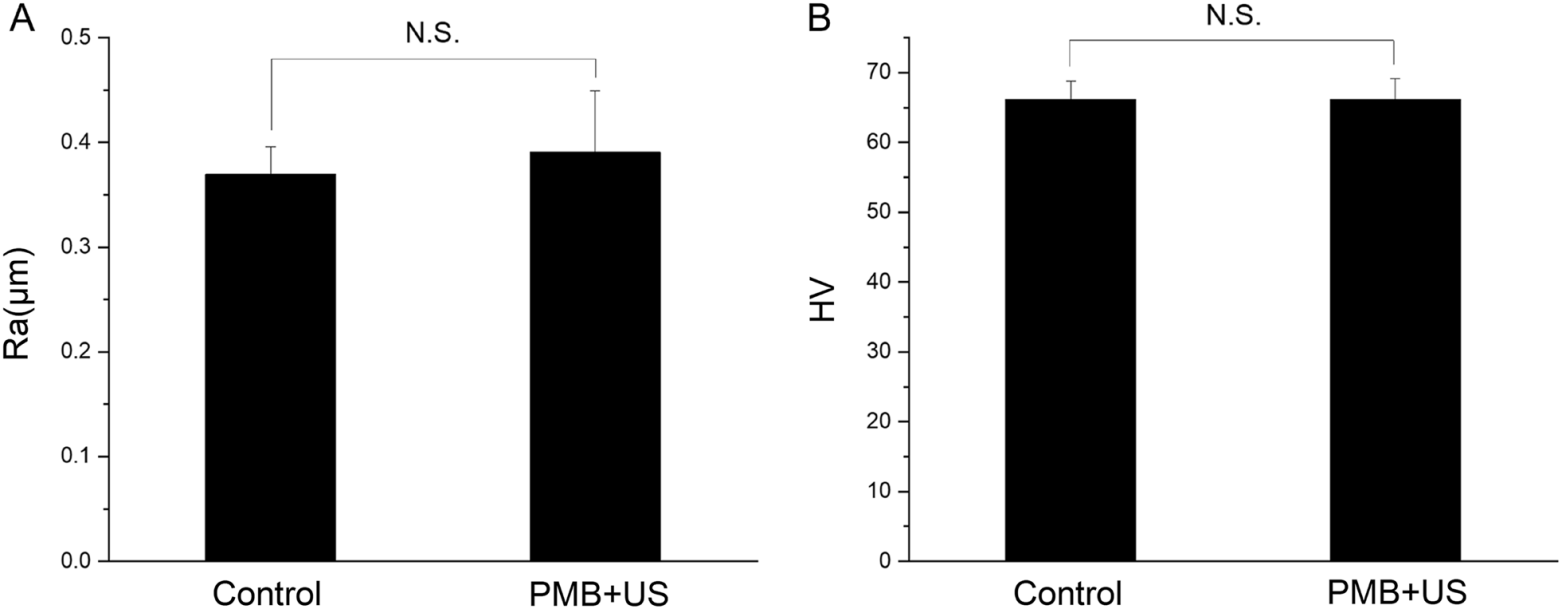
Roughness (A, Ra, μm) and microhardness (B, HV) of dentin before and after PMBs + US treatment. “N.S.” means not significant (*p* > 0.05)

As Fig. 7 shows, the roughness (A, Ra) and microhardness (B, HV) of dentin block were 0.3694 μm and 66.1667, respectively. After treated with PMBs + US, the results were 0.3906 μm and 66.1733, respectively. Obviously, there are no significant differences in roughness and microhardness after PMBs treatment (*p* > 0.05). This indicates the treatment with PMB + US will not change the mechanical properties of dentin.

## Discussion

The most crucial objective of root canal treatment is to minimize the number of microorganisms and pathologic debris in canal systems to prevent or treat apical periodontitis. However, it is hard to achieve clinically due to the anatomical complexity of the root canal system and the innate resistance of biofilms. *E. faecalis* possesses some special abilities to contribute to its persistent existence, such as surviving in harsh environment, invading dentin tubules and adhere to dentine and to resist to common disinfectants [1]. Commonly used root canal irrigations like NaOCl and CHX have been recognized antibacterial effect on *E. faecalis*, but there are deficiencies such as the toxicity of NaOCl solution and negligible ability of CHX to remove biofilm [26, 27]. Meanwhile, ultrasonic irrigation of the root canal has been reported to be more efficient in debris debridement, irrigants distribution in canal system and bacteria removal [28].

Cold atmosphere pressure plasma has promising potential for various medical and biomedical applications. Our previous study had demonstrated that plasma shows great antibacterial effect against *E. faecalis* in mature biofilm [19]. Although cold plasma has antibacterial effect against cells inside, there still remained some debris and fused cell bodies on the surface of root canal. Microbubbles have been widely applied in diagnostic medicine as ultrasound contrast agents. More important, microbubbles have been reported to be a suitable carrier for drugs. The PMBs, which could be a vehicle to delivery and release plasma and prolong the effective time of reactive species from plasma. This newly proposed method was expected to combine the antibacterial capacity of plasma and the cavitation effect of microbubbles after ultrasound exposure.

As shown in Fig. 3, the stable micrometer size enables PMBs release into narrow canal system and even dentine tubules. The volume stability reflects that it can preserve and prolong the effective time of reactive substance in the plasma. In order to verify whether microbubbles encapsulated reactive species from plasma and to interpretation the potential inactivation mechanism, we measured the concentration of NO and H_2_O_2_, which are highly related to bactericidal effects [29, 30]. Before sonication, the concentration of reactive substances in PMBs was significantly higher than that in the control group (PBS). Meanwhile, a portion of plasma gas was encapsulated in the microbubbles formed. Once exposed to ultrasound, the microbubbles could undergo oscillation and collapse, accompanied by the release of the plasma. Thus, the NO and H_2_O_2_ concentration increased remarkably after ultrasound treatment.

To evaluate the disinfection effect, *E. faecalis* biofilm on confocal dishes were cultivated for 7 days. CLSM analysis showed that PMBs with ultrasound exposure presented great removal effect against biofilm compared with PBS group and CHX group. The removal effect of PMBs group were superior to 2.5% NaOCl group. Additionally, the bacteria elimination effect in high concentration group (10^8^ mL^−1^) was more efficient compared with NaOCl group (Fig. 4E, B). Meanwhile, the remaining bacteria on the dishes were also been killed (red fluorescence) compared with PBS group (green fluorescence). Thus, PMBs with the assistance of ultrasound exhibit excellent antimicrobial effect and biofilm removal effect.

To further verify the removal effect of PMBs, the 7d *E. faecalis* biofilm on human dentin blocks were observed. Biofilm formation generally comprises several stages like initial attachment, microcolony formation and biofilm maturation [31]. Several studies have demonstrated that “young” biofilm could be formed when cultivated for 7 days and grow mature as incubation time prolonged. The SEM results demonstrated that PMBs with ultrasound treatment could effectively remove the bacteria and extracellular matrix (Fig. 6D, E). Compared with NaOCl group, it was more effective against bacteria inside the dentin tubules (Fig. 6B). The NaOCl solution cannot invade into narrow dentin tubules because of the high surface tension and low wettability, so the bacteria and biofilm components still live in the dentin tubules [32]. Although the similar removal effect had been observed on confocal dishes (Fig. 4B vs. D and E), the PMBs could effectively “dig out” those bacteria and extracellular matrix embedded in dentin tubules. As Fig. 6D, E show, the dentin tubules were clean and intact, but the dentin surface was corroded with 2.5% NaOCl (Fig. 6B). Thus, PMBs with ultrasound treatment could be an effect biofilm removing methods especially for those biofilms hidden in the dentin tubules and benefit to the following canal obturation procedure.

As to bactericidal and biofilm removal mechanisms, NO is one of the primary biological active species in plasma and an important membrane-permeable signal gas molecule that has been implicated in a wide range of physiological process [33]. It has been confirmed that NO is toxic to bacteria at high concentrations (~ μM). While relatively low concentrations (nM–μM) of NO appears to be non-toxic and a quorum sensing signal that mediate physiological responses like biofilm dispersal [33]. H_2_O_2_ could cause oxidative damage to all living substances like nucleic acid, protein and lipid and resulting in cell death [30]. It is acknowledged that the working concentration of sole H_2_O_2_ usually reaches up to 900–3000 mmol/L [34]. Hence the inactivation effect of PMBs with ultrasound is presumably attributed to the synergistic effect of chemical reaction of NO and the mechanical effect of ultrasonic cavitation. The biofilm removal effect and antibacterial ability of PMBs + US could make it a potential method for infection control in narrow spaces and complex anatomical structures, such as around the implant, in the deep periodontal pocket, in root canals, even within the dentine tubules.

In present study, PMBs with ultrasound treatment exhibited equally excellent result as NaOCl. Reduction in microhardness of dentin were reported when 1–6% NaOCl were used for irrigation during endodontic therapy [35, 36]. It is proved that the PMBs with ultrasound treatment did not significantly change the microhardness and roughness of dentin blocks. The PMBs are mechanically safe when applied in endodontics treatment.

In conclusion, PMBs combined with ultrasound has been demonstrated to act as an innovative and effective endodontic disinfection method for its excellent effectiveness and safety. In this study, an ultrasound device with a probe was used to emit ultrasonic waves, which is totally different from traditional ultrasonic files in clinic. The ultrasound waves could propagate through the liquid medium and stimulate the cavitation of PMBs. The operating mode is not the mechanical vibration with traditional ultrasonic files, but the enhanced cavitation due to the microbubbles which could act as crystallization nuclei. More important, this method could avoid the occurrence of ultrasonic files separation completely. In addition, there are some limitations in present study: (1) trials in root canal system and in vivo experiment should be done; (2) the cultivation time should be prolonged to form mature biofilm; (3) the quantitative experiments are needed to assess the amount of biofilm; (4) to further explore the relevant mechanisms.

## Conclusion

PMBs combined with ultrasound is a promising method for endodontic applications for the strong biofilm removal effect and significant antibacterial effect against *E. feacalis*, together with its satisfactory mechanical safety on tooth hard tissue.

## Data Availability

The datasets used and/or analyzed during the current study are available from the corresponding author on reasonable request.

## Abbreviations

PMBs: Plasma-loaded microbubbles
*E. faecalis*: *Enterococcus faecalis*
NaOCl: Sodium hypochlorite
CHX: Chlorhexidine
Ca (OH)_2_: Calcium hydroxide
PDT: Photodynamic therapy
Qmix: A root canal irrigation mixture of ethylenediamine tetraacetic acid, chlorhexidine and surfactant
MTAD: A root canal irrigation mixture of doxycycline, citric acid and detergent
PMBs: Plasma-loaded microbubbles
NO: Nitric oxide
H_2_O_2_: Hydrogen peroxide
CEJ: Cementoenamel junction
EDTA: Ethylene diamine tetraacetic acid
PBS: Phosphate buffer solution
BHI: Brain heart infusion
US: Ultrasound
CLSM: Confocal laser scanning microscopy
SEM: Scanning electron microscopy
Ra: Roughness
VH: Vivkers hardness
ANOVA: An analysis of variance
